# Common opportunistic infections and their CD4 cell correlates among HIV-infected patients attending at antiretroviral therapy clinic of Gondar University Hospital, Northwest Ethiopia

**DOI:** 10.1186/1756-0500-6-534

**Published:** 2013-12-14

**Authors:** Debasu Damtie, Gizachew Yismaw, Desalegn Woldeyohannes, Belay Anagaw

**Affiliations:** 1 Department of Immunology and Molecular biology, School of Biomedical and Laboratory Sciences, College of Medicine and Health Sciences, University of Gondar, Gondar, Ethiopia; 2 Department of Medical Microbiology, School of Biomedical and Laboratory Sciences, College of Medicine and Health Sciences, University of Gondar, Gondar, Ethiopia; 3 Department of Public Health, School of Medical and Health Sciences, Addis Ababa Science and Technology University, Addis Ababa, Ethiopia

**Keywords:** CD4 correlates, Ethiopia, Gondar, HIV-infected patients, Opportunistic infections

## Abstract

**Background:**

Human immunodeficiency virus (HIV) pandemic is among the greatest health crises ever faced by humanity. Morbidity and mortality in HIV disease is due to immunosuppression leading to life-threatening opportunistic infections (OIs) during the natural course of the disease. This study was aimed to assess the prevalence and CD4 correlates of OIs among adult HIV-infected patients attending at Gondar University Hospital.

**Methods:**

Cross sectional study was conducted on 360 adult HIV-infected patients attending antiretroviral therapy clinic from February 2012-April 2012. Patients’ OI status was determined through clinical diagnosis and laboratory investigations. CD4 count was determined using flow cytometry technique. Sociodemographic and clinical data were obtained from interview and patients’ medical records. Bivariate and multivariate logistic regression analysis was done using SPSS version 16 statistical soft ware and odds ratio was used as the measure of association. P-value less than 0.05 was considered statistically significant for all tests.

**Results:**

In this study, 360 HIV-infected patients were included; of whom (n = 216/360, 60%) were females. The majority of patients (n = 153/360, 42.5%) were 25-34 years old with mean age of 35.5+ 8.8 standard deviation. The overall prevalence of OIs was (n = 71/360, 19.7%). Tuberculosis (n = 35/360, 9.72%) followed by oral candidiasis (n = 18/360, 5%) and diarrhea (n = 12/360, 3.3%) were the most frequently observed OIs. CD4 count less than 200/mm^3^ (OR = 4.933, P < 0.001), World Health Organization (WHO) clinical stage III (OR = 9.418, P < 0.001) and IV (OR = 22.665, P < 0.001) were found to have strong association with acquisition of OIs.

**Conclusions:**

Tuberculosis, oral candidiasis and diarrhea were the leading OIs encountered by HIV-infected patients. CD4 count less than 200/mm^3^ and advanced WHO clinical stages of the disease were found to be predictors of OIs. Interventions aimed at preventing and treating HIV associated OIs are crucial. Initiation of ART before the CD4 count drops below 350 should be encouraged.

## Background

Human Immunodeficiency Virus (HIV) pandemic is among the greatest health crises ever faced by humanity. Globally, 34.0 million people were living with HIV at the end of 2011 [1]. Sub-Saharan Africa remains most severely affected, with nearly 1 in every 20 adults (4.9%) living with HIV [1]. In Ethiopia, approximately one million people are living with HIV which has become the leading cause of mortality among 15-49 years of age, that accounts for about 43% of all population in 2008 [2].

Morbidity and mortality in HIV disease result due to underlying immunosuppression which leads to life-threatening opportunistic infections (OIs) during the natural course of the disease [3]. the widespread use of ART starting in the mid-1990s has had the most profound influence on reducing OI-related mortality in HIV-infected persons in those countries in which these therapies are accessible and affordable [4,5]. However, OIs continue to cause morbidity and mortality in HIV/AIDS patients even after ART. Some patients do not have a sustained response to antiretroviral agents for multiple reasons including poor adherence, drug toxicities, drug interactions, or initial acquisition of a drug-resistant strain of HIV-1. Therefore OIs continue to cause substantial morbidity and mortality in patients with HIV-1 infection despite use of ART [6].

Despite the fact that different studies have been conducted on the prevalence of individual OIs among HIV-infected patients in developing countries like Ethiopia, information about the magnitude and spectrum of OIs and their CD4 correlates is scarce in the study area. Therefore, this study was aimed to add updates to the existing data on extent of OIs and their immunological correlates among HIV-infected patients attending ART clinic of the Gondar University Hospital.

## Methods

### Study design and period

Institution based cross sectional study was conducted among adult HIV-infected patients attending at ART clinic of University of Gondar Hospital from February 2012-April 2012.

### Study population and study area

The study population was all registered adult HIV-infected patients attending ART clinic of Gondar University Hospital for medical attention and consultation during the study period. Study participants were screened for OIs only once during the study period. The study was conducted at the Gondar University Hospital ART clinic. The University Hospital is a teaching Hospital situated in Gondar town, 737 Km away from the capital Addis Ababa with a projected population of 248,784 (Zonal statistics office). The Hospital gives different inpatient and outpatient services to the population in the surrounding area of Gondar town and the adjacent regions. There is ART clinic in the Hospital which provides voluntary counseling and testing (VCT), ART and diagnostic services. All HIV-infected patients attending the clinic are assessed for OIs. Moreover patients are regularly followed for immunological, organ function and hematological tests.

### Variables

Socio demographic characteristics, CD4 count, Prophylaxis history and WHO clinical stage of HIV-infected patients were considered as explanatory variables; while opportunistic infection status of HIV-infected patients was considered as outcome variables of this study.

### Sample size and sampling techniques

Sample size was calculated using single population proportion formula [7]. A total of 360 HIV-infected patients were included in the study considering 5% non response rate. Study participants were selected through systematic random sampling method among HIV-infected patients visiting the ART clinic during the study period.

### Data collection procedure

#### Sociodemographic characteristics and other factors

Sociodemographic and other relevant data were collected by nurses through interview and from patients’ medical records using structured data collection format. Orientation and supportive supervision were given to data collectors to maintain data quality.

#### Diagnosis of OIs

The diagnosis of the diseases was done through clinical examination and laboratory investigation by two physicians and one Health Officer in charge of examining patients at ART outpatient department as per Federal Ministry of Health guideline for management of OIs and ART [8]. The patients who had more than two weeks of cough were considered for the tuberculosis examination. The diagnosis of tuberculosis (TB) was confirmed by microscopic examination of the sputum using acid fast bacilli (Ziehl- Neelsen) staining followed by chest radiogram when negative for microscopy. Diagnosis of Extra Pulmonary Tuberculosis (EPT) was done through physical examination followed by fine needle aspiration (FNA) smear microscopy for acid fast bacilli (AFB) and cytological examination. For the conformation of chronic diarrhea, solely clinical presentations and stool wet mount were considered and no other types of investigations pertaining to the causal agents were investigated. Oral candidiasis, skin fungal infections and other OIs were diagnosed by clinical presentations of patients.

#### CD4+ cell count

The CD4+ cell count of HIV seropositive persons were estimated using FACSCalibur flow cytometer (Becton Dickinson, California, USA). The results of CD4 count was reported as absolute count of CD4 cells/mm^3^ of blood from the FACSCalibur CD4 counting machine.

### Data processing and analysis

The data was entered and analyzed using SPSS (version 16) statistical soft ware. Logistic regression analysis was done for variables significant at bi-variant analysis and odds ratio was used as the measure of association between the dependent variable and the co-variants. P-value less than 0.05 was considered statistically significance for all tests.

### Data quality management and ethical consideration

Federal Ministry of Health guideline for management of OIs and ART was strictly followed during clinical assessment of OIs to maintain data quality. The CD4 count was done after running control beads to check whether the CD4 counting machine was working properly or not. Moreover data collector nurses were given supportive supervision to maintain data quality.

Ethical approval was obtained from the School Research and Ethical Review Committee, College of Medicine and Health Sciences, University of Gondar. All OI positive patients were given treatment at the ART clinic as per the OI treatment guideline.

## Results

### Sociodemographic and clinical characteristics

In this study, 360 adult HIV-infected patients were included; of whom (n = 216/360, 60%) were females. The majority of patients (n = 153/360, 42.5%) were in age group 25-34. The mean age of the study subjects was 35.5+ 8.8 standard deviation ranging from 19 to 72. According to CD4 cell count/mm^3^ of blood, majority (n = 187/360, 51.9%) had CD4 count of >350 cells/mm^3^. Majority of the study subjects were WHO clinical stage I (n = 273/360, 75.8%) while least number of study subjects were WHO clinical stage IV (n = 17/360, 4.7%). Most of the study subjects were on ART and cotri-moxazole preventive therapy which account (n = 281/360, 78%) and (n = 301/360, 83.6%) respectively [Table 1].

**Table 1:** **Socio demographic characteristics and logistic regression analysis of risk factors for OIs among HIV**-**infected patients attending at ART clinic of Gondar University Hospital**, **February**- **April 2012**

**Characteristics**	**N (%)**	**OIs**		**COR (P-value)**	**AOR (P-value)**
		**Yes (%)**	**No (%)**		
**Age**					
15-24	28 (7.8)	7 (25)	21 (75)	1.00	1.00
25-34	153 (42.5)	30 (19.6)	123 (80.4)	0.732 (0.517)	0.749 (0.649)
35-44	123 (34.2)	25 (20.3)	98 (79.7)	0.765 (0.568)	0.536 (0.358)
45-54	45 (12.5)	6 (13.3)	39 (86.7)	0.462 (0.211)	0.363 (0.231)
>/=55	11 (3.1)	3 (27.30)	8 (72.7)	1.125 (0.884)	0.317 (0.327)
**Marital status**					
Single	65 (18.1)	20 (30.8)	45 (69.2)	1.00	1.00
Married	144 (40)	19 (13.20)	125 (86.8)	0.342 (0.003)*	0.445 (0.104)
Divorced	86 (23.9)	25 (29.1)	61 (70.9)	0.922 (0.821)	1.428 (0.492)
Separated	10 (92.7)	0 (0)	10 (100)	_	_
Widow	55 (15.3)	7 (12.7)	48 (87.30	0.328 (0.022)*	0.698 (0.492)
**CD4 count (cells/mm**^**3**^**)**					
<200	71 (19.7)	41 (57.7)	30 (42.3)	14.602 (<0.001)*	4.933 (<0.001)*
200-350	102 (28.3)	14 (13.7)	88 (86.3)		1.173 (0.723)
>350	187 (52)	16 (8.6)	171 (91.4)	1.7 (0.172)	1.00
**WHO clinical stage**					
I	273 (75.8)	19 (7)	254 (93)	1.00	1.00
II	19 (5.3)	6 (31.6)	13 (68.4)	6.17 (0.001)*	3.005 (0.098)
III	51 (42.2)	32 (62.7)	19 (37.3)	22.517 (<0.001)*	9.418 (<0.001)*
IV	17 (4.7)	14 (82.4)	3 (17.6)	62.384 (<0.001)*	22.665 (<0.001)*
**Co**-**trimoxazole prophylax**					
Yes	301 (83.6)	47 (15.6)	254 (84.4)	1.00	1.00
No	59 (16.4)	24 (40.7)	35 (59.3)	3.706 (<0.001)*	1.106 (0.865)
**ART status**					
Pre-ART	79 (22)	35 (44.3)	44 (55.7)	3.414 (<0.001)*	2.0003 (0.093)
On-Art	281 (78)	36 (12.8)	245 (87.2)	1.00	1.00

*Statistically significant association, *COR* = Crude Odds Ratio, *AOR* = Adjusted Odds Ratio.

### Prevalence of OIs

**Figure 1:**
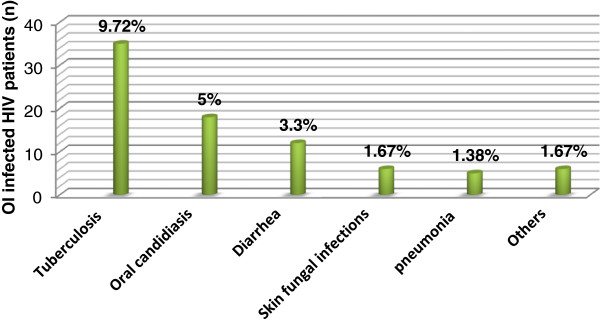
**Common OIs at ART clinic of Gondar University Hospital, February-April 2012.** Others: Acute herpes zoster attack, Acute tonsillopharyngitis, Kaposi’s sarcoma and Molluscum contagiosum.

The overall prevalence of OIs was found to be (n = 71/360, 19.7%). Tuberculosis emerged as the most frequent infection to be associated with HIV infection in patients across the total range of CD4+ followed by oral candidiasis among the spectrum of OIs observed. Tuberculosis infection was found to be prevalent among (n = 35/360, 9.72%) of patients. Of these (n = 30/35, 85.71%) were pulmonary tuberculosis while extra pulmonary types account (n = 5/35, 14.29%). Majority (n = 22/30, 73.33%) of the pulmonary tuberculosis patients were smear negative while (n = 8/30, 26.66%) were smear positive pulmonary TB patients. Oral candidiasis emerged as the second most prevalent infection (n = 18/360, 5%) followed by diarrheal diseases, skin fungal infection, pneumonia and others with prevalence of (n = 12/360, 3.3%), (n = 6/360, 1.67%), (n = 5/360, 1.38%) and (n = 6/360, 1.67%) respectively [Figure 1]. Among diarrheic patients (n = 3/12, 25%) were positive for parasitic investigations, two were positive for larva of *Strongyloid stercolaris* and one positive for ova of *Schistosoma mansonia*. There were twelve co-infections of different OIs observed [Table 2].

**Table 2:** Co-infection of different OIs at ART clinic of Gondar University Hospital, February-April 2012

**Co**-**infection of OIs**	**Frequency (%)**
TB and oral candidiasis	6 (50)
TB and skin fungal infection	1 (8.33)
Diarrhea and oral candidiasis	2 (16.66)
Skin fungal infection and oral candidiasis	2 (16.66)
Diarrhea, oral candidiasis and skin fungal infection	1 (8.33)
**Total**	**12 (100)**

### Opportunistic infections and CD4 levels

The prevalence of OIs was found to be highest (n = 41/71, 57.7%) among HIV-infected patients with CD4 count less than 200/mm^3^ followed by CD4 count 200-350/mm^3^ and count above 350/mm^3^ with prevalence of (n = 14/102, 13.3%) and (n = 16/187, 8.6%) respectively. CD4 count less than 200/mm^3^ is found to have strong association with acquisition of OIs (OR = 4.933, P < 0.001) [Table 1]. Higher prevalence of tuberculosis (n = 21/71, 29.58%), diarrhea (n = 4/71, 5.63%), oral candidiasis (n = 16/71, 22.53%), skin fungal infections (n = 4/71, 5.63%), pneumonia (n = 3/71, 4.22%) and others (n = 4/71, 5.63%) were found in patients with CD4 count less than 200/mm^3^. Each OI diagnosed showed strong association with CD4 count less than 200/mm^3^; oral candidiasis (OR = 27.20, P < 0.001), tuberculosis (OR = 9.40, P < 0.001), skin fungal infections (OR = 11.10, P = 0.008) and pneumonia (OR = 8.21, P = 0.032) [Table 3].

**Table 3:** **Association of CD4 level with OIs at ART clinic of Gondar University Hospital**, **February**- **April 2012**

**Type of OIs**	**Positive N (%)**	**Negative N (%)**	**OR (P-value)**
Tuberculosis			
<200	21 (29.58)	50 (70.42)	9.40 (<0.001)*
200-350	6 (5.88)	96 (94.22)	1.40 (0.544)
>350	8 (4.28)	179 (95.72)	1.00
Total	35 (9.72)	325 (90.28)	
Diarrhea			
<200	4 (5.63)	67 (94.37)	3.66 (0.075)
200-350	5 (4.90)	97 (95.10)	3.16 (0.103)
>350	3 (1.60)	184 (98.40)	1.00
Total	12 (3.33)	348 (96.77)	
Oral candidiasis			
<200	16 (22.53)	55 (77.47)	27.20 (<0.001)*
200-350	0 (0.00)	102 (100)	_
>350	2 (1.06)	185 (98.94)	1.00
Total	18 (5.00)	342 (95.00)	
Skin fungal infections			
<200	4 (5.63)	67 (94.37)	11.10 (0.008)*
200-350	1 (0.98)	101 (99.02)	1.84 (0.662)
>350	1 (0.53)	186 (99.47)	1.00
Total	6 (1.67)	354 (98.33)	
Pneumonia			
<200	3 (4.22)	68 (95.78)	8.21 (0.032)*
200-350	1 (0.98)	101 (99.02)	1.84 (0.662)
>350	1 (0.53)	186 (99.47)	1.00
Total	5 (1.38)	355 (98.62)	
Others			
<200	4 (5.63)	67 (94.37)	11.10 (0.008)*
200-350	1 (0.98)	101 (99.02)	1.84 (0.662)
>350	1 (0.53)	186 (99.47)	1.00
Total	6 (1.67)	354 (98.33)	

*Statistically significant association, *OR* = Odds ratio.

### Associated risk factors of OIs

The prevalence of OIs was comparable (p = 0.482) among males (n = 31/144, 21.5%) and females (40/216, 18.5%). Although no statistically significant association was observed (P = 0.721), highest prevalence of OIs was depicted among age group 55 and above (n = 3/11, 27.3%). As far as marital status is concerned single and divorced study subjects depicted higher prevalence (n = 20/65, 30.8%) and (n = 25/86, 29.1%) respectively (P = 0.001). Almost similar prevalence of OIs was observed among different educational levels (P = 0.977) [Table 1].

World Health Organization clinical stage VI and III showed highest prevalence (n = 14/17, 82.4%) and (n = 32/51, 62.7%) while least prevalence was observed among clinical stage I patients (n = 19/273, 7%). Statistically significant association was depicted between prevalence of OIs and WHO clinical stage III (OR = 9.418, P < 0.001) and IV (OR = 22.665, P < 0.001). Prevalence of OIs was higher (OR = 1.106, P = 0.865) among patients who were not on co-trimoxazole prophylaxis (n = 24/59, 40.7%) compared to their counter parts who were on co-trimoxazole prophylaxis (n = 47/301, 15.6%). Similarly the prevalence of OIs was higher (OR = 2.003, P = 0.093) in ART-naïve patients (n = 35/79, 44.3%) as compared to patients exposed to ART (n = 36/281, 12.8%). However, the difference was not statistically significant [Table 1].

## Discussion

Although HIV is the initial causative agent for AIDS, most of the morbidity and mortality seen in immunocompromised patients results from OIs that take advantage of the lowered cellular and humoral defense of the patient. The overall prevalence of OIs in the present study was (n = 71/360, 19.7%) which is a bit lower than a 27.4% reported prevalence of OIs among new adult AIDS cases at New York City Department of Health and Mental Hygiene [9]. The difference in this finding might be due to the difference in study subjects; the study in New York City included symptomatic AIDS patients while this study included both healthy and symptomatic patients which possibly reduce the prevalence of OIs in the present study. The finding is by far lower than studies conducted in Kolkata, India and Bahir Dar, Ethiopia which documented 53.4% and 88.9% prevalence respectively [10,11]. This difference might be explained by methodological differences in selecting study subjects in case of Bahir Dar city and difference in ascertainment of OIs in case of the study in India.

The present study revealed that tuberculosis infection followed by oral candidiasis and diarrheal diseases emerged as the predominant OIs identified with prevalence of (n = 35/360, 9.72%), (n = 18/360, 5%) and (n = 12/360, 3.3%) respectively. According to WHO’s 2007 report, the prevalence of all forms of TB in Ethiopia was estimated at 546 per 100,000 populations; which is far lower than the finding of this study [12]. This implies that TB infection is opportunistic among HIV-infected people even in high TB burden countries including Ethiopia. This finding is in line with studies from India which revealed tuberculosis as a major OI identified [13-15]. However another study conducted in India revealed that oral candidiasis (53.43%) followed by chronic diarrhea (47.05%) are the commonest OIs identified while TB stands fourth with the prevalence of 35.29% [10]. Despite the similarity in the order of OIs in different studies there is still a disparity in the magnitude of each OI; this might possibly be explained by differences in the prevalence of OIs in the general population or differences in the methodology and OI screening criteria used.

There were twelve co-infections of different OIs observed. Of these, (n = 6/12, 50%) were tuberculosis and oral candidiasis co-infections. Higher proportion of TB and OC co-infection in this study might be explained by higher prevalence rate of these two OIs among the study subjects. Dual and triple OIs were also reported from study in India [10].

The hospital where this study was conducted initiates antiretroviral therapy when the CD4 level of a patient becomes below 200/mm^3^ of blood which is far lower than the recommendation by WHO which increase susceptibility of HIV-infected individuals to OIs. In this study HIV-infected patients with CD4 count less than 200/mm^3^ were found to be 4.9 times more likely to develop OIs compared to the reference group patients with CD4 count >350/mm^3^. This finding is concordant with other studies from India which reported high risk of developing OIs such as TB, *Pneumocystis jiroveci* pneumonia, and *cryptococcal* meningitis among patients with CD4 counts <200 cells/mm^3^[16-18]. The patients’ CD4 count was also found to be associated with development of individual OIs. Patients with CD4 count less than 200/mm^3^ are 9.4, 27.2, 11.1 and 8.2 times more likely to develop tuberculosis, oral candidiasis, skin fungal infections and pneumonia respectively compared to the reference category CD4 count >350/mm^3^. This finding sounds true since CD4 cells play a central role in the activation of both humoral and cellular immune response to fight against infection. Hence, low CD4 count increases susceptibility to OIs.

World Health Organization clinical stage III and IV HIV-infected patients were 9.4 and 22.6 times more likely to develop OIs compared to clinical stage I counter parts respectively. This finding is in agreement with the studies from India and South Africa which depicted that advanced clinical stage of the disease is significantly associated with development of OIs among patients on ART [19-21].

### Limitations

Since the Hospital where this study was conducted does not routinely perform culture for the diagnosis of OIs due to unavailability of culture for OIs, this study was limited to identify etiology of most of the OIs. Hence majority of the OIs were screened clinically which may affect the diagnostic accuracy.

## Conclusions

In the presence of all diagnostic limitations mentioned above, the overall frequency of OIs in this study was significant. Tuberculosis followed by OC and diarrhea were the major OIs encountered by HIV-infected patients. CD4 count less than 200/mm^3^ and WHO clinical stage III and IV were found to be strongly associated with prevalence of OIs. Interventions aimed at preventing and treating HIV associated OIs is crucial. Commencement of ART should be encouraged before the patients’ CD4 count drops below 350/mm^3^ since the local practice is different from the WHO’s recommendation for the commencement of ART which is CD4 count <200/mm^3^.

## Abbreviations

AFB: Acid fast bacilli; AIDS: Acquired immunodeficiency syndrome; ART: Antiretroviral treatment; CD: Cluster of differentiation; CMV: Cytomegalovirus; CNS: Central nervous system; EPTB: Extra pulmonary tuberculosis; FNA: Fine needle aspiration; GUH: Gondar University Hospital; HAART: Highly active antiretroviral treatment; HIV: Human immunodeficiency virus; HSV: Herpes simplex virus; IRIS: Immune reconstitution inflammatory syndrome; MTB: *Mycobacterium tuberculosis*; OC: Oral candidiasis; OIs: Opportunistic infections; PVL: Plasma viral load; SOP: Standard operating procedure; TB: Tuberculosis; VCT: Voluntary counseling and testing; WHO: World Health Organization.

## Competing interests

The authors declare that they have no competing interests.

## Authors’ contributions

DD carried out the proposal writing, participated in the data collection, CD4 count, data analysis and drafted the manuscript. DD, GY, DW and BA were participated in the final write up of the paper, data analysis and interpretation of the findings. DD: responsible for drafting the manuscript. All authors were involved in reviewing the manuscript and approval for publication.

## References

[B1] UNAIDS Report on the Global AIDS Epidemic 2012 Geneva: UNAIDS

[B2] Federal Ministry of Health (FMoH) Annual HIV/AIDS monitoring and evaluation report in Ethiopia 2009 Ethiopia, Addis Ababa: Federal Ministry of Health

[B3] Nissapatorn V Lee C Fatt QK Abdullah KA AIDS-related opportunistic infections in Hospital Kuala Lumpur Japan J of Infectious Dis 2003 56 5–6 187 192 14695428

[B4] Miller V Mocroft A Reiss P Relations among CD4 lymphocyte count nadir, antiretroviral therapy, and HIV-1 disease progression: results from the Euro SIDA study Annual Inter Med 1999 130 570 577 10.7326/0003-4819-130-7-199904060-00005 10189326

[B5] Dore GJ Li Y McDonald A Ree H Kaldor JM Impact of highly active antiretroviral therapy on individual AIDS-defining illness incidence and survival in Australia J Acquired Immune Def Synd 2002 29 388 395 10.1097/00126334-200204010-00010 11917244

[B6] Federal HIV/AIDS prevention and control office federal ministry of health Guidelines for management of opportunistic infections and antiretroviral treatment in adolescents and adults in Ethiopia 2007 http://www.ilo.org/wcmsp5/groups/public/---ed_protect/---protrav/---ilo_aids/documents/legaldocument/wcms_125386.pdf

[B7] Cochran WG Sampling Techniques 1963 2 New York: John Wiley and Sons Inc 75

[B8] Federal Ministry of Health Guidelines for management of opportunistic infections and anti retroviral treatment in adolescents and adults in Ethiopia 2008 http://www.who.int/hiv/pub/guidelines/ethiopia_art.pdf

[B9] Hanna DB Gupta LS Jones LE AIDS-defining opportunistic illnesses in the HAART era in New York City Acquired Immune Def Synd Care 2007 19 2 264 272 10.1080/09540120600834729 17364409

[B10] Kallol S Rushna F Poonam S Recent pattern of Co-infection amongst HIV seropositive individuals in tertiary care hospital Kolkata. Virology J 2011 8 116 10.1186/1743-422X-8-116 PMC3066117 21396133

[B11] Bayeh A Fisseha W Tsehaye T Atnaf A Mohammed Y ART-naive HIV-infected patients at Feleg-Hiwot Referral Hospital Northwest, Ethiopia Ethiopian J of Health Dev 2010 24 1 3 8

[B12] WHO Global tuberculosis control report 2007 Geneva: WHO Press http://www.afro.who.int/index.php?option=com_docman&task=doc_download&gid=483

[B13] Vajpayee M Kanswal S Seth P Wig N Spectrum of Opportunistic Infections and Profile of CD4+ Counts among AIDS Patients in North India J Infection 2003 31 336 340 10.1007/s15010-003-3198-y 14556060

[B14] Manisha G Swapna D Srikanth T Madhura N Preeti G Incidence of common opportunistic infections in HIV-infected individuals in Pune, India: analysis by stages of immunosuppression represented by CD4 counts Inter J In Dis 2009 13 1 8 10.1016/j.ijid.2008.03.029 18602329

[B15] Dominic S Nitika G Shalini S Vishwas S Prevalence of opportunistic infections in AIDS patients in Mangalore, Karnataka J Trop Doctors 2008 38 3 172 173 10.1258/td.2007.070171 18628550

[B16] Simon M Joseph O Fred S Factors associated with development of opportunistic infections among patients on ART at a Ugandan Program-MJAP. From 16th International Symposium on HIV and Emerging Infectious Diseases Marseille, France J Retrovirology 2010 7 1 77 10.1186/1742-4690-7-77

[B17] Robin W Gary M Carl JL Risk factors for developing tuberculosis in HIV-1–infected adults from communities with a low or very high incidence of tuberculosis J Acquired Immune Def Synd 2000 23 75 80 10.1097/00126334-200001010-00010 10708059

[B18] WHO Guidelines for HIV diagnosis and monitoring of antiretroviral therapy 2005 Geneva: WHO Press

[B19] Gautam H Bhalla P Saini S Epidemiology of opportunistic infections and its correlation with CD4 T-lymphocyte counts and plasma viral load among HIV-positive patients at a tertiary care hospital in India J Inter Asso Physicians in Acquired Immune Def Synd Care 2009 8 6 333 337 10.1177/1545109709346881 19755619

[B20] Badri M Wilson D Wood R Effect of highly active antiretroviral therapy on incidence of tuberculosis in South Africa: a cohort study Lancet 2002 359 2059 2064 10.1016/S0140-6736(02)08904-3 12086758

[B21] Kumarasamy N Vallabhaneni S Flanigan TP Mayer KH Solomon S Clinical profile of HIV in India Indian J Med Res 2005 121 4 377 394 15817951

